# A Safe and Effective Atovaquone-Proguanil Therapeutic Protocol for the Treatment of Avian Malaria by *Plasmodium relictum* in Snowy Owl (*Bubo scandiacus*)

**DOI:** 10.3390/ani13223457

**Published:** 2023-11-09

**Authors:** Nicola Pugliese, Rossella Samarelli, Roberto Lombardi, Antonella Schiavone, Giuseppe Crescenzo, Elena Circella, Claudia Zizzadoro, Olimpia Lai, Medhat S. Saleh, Michela Prioletti, Antonio Camarda

**Affiliations:** Dipartimento di Medicina Veterinaria, Università degli Studi di Bari, 70010 Valenzano, Italy; rossella.samarelli@uniba.it (R.S.); roberto.lombardi@uniba.it (R.L.); antonella.schiavone@uniba.it (A.S.); giuseppe.crescenzo@uniba.it (G.C.); elena.circella@uniba.it (E.C.); claudia.zizzadoro@uniba.it (C.Z.); olimpia.lai@uniba.it (O.L.); medhat.elshahat@uniba.it (M.S.S.); michela.prioletti@gmail.com (M.P.); antonio.camarda@uniba.it (A.C.)

**Keywords:** avian malaria, *Plasmodium relictum*, *Bubo scandiacus*, efficacy safety, atovaquone, proguanil, antiprotozoal

## Abstract

**Simple Summary:**

This study assessed the efficacy of treating snowy owls naturally infected by *Plasmodium relictum* with atovaquone (AV) and proguanil (PH). AV/PH was administered at 10/4 mg/kg/day for three days, repeated a week later. Results showed the treatment was effective in clearing the parasite from the birds’ blood, with no relapses observed. Additionally, hematological improvements were noted, suggesting recovery. No significant adverse effects were observed, indicating the safety of the treatment. This study highlights the potential of AV/PH as a treatment option for avian malaria in unconventional or endangered bird species.

**Abstract:**

Avian malaria is a re-emerging threat to avian species worldwide. It is sustained by several protozoan species belonging to the genus *Plasmodium*, mainly *Plasmodium relictum*. The even wider diffusion of the disease, probably because of the increase in the areas covered by their mosquito vectors, may pose new risks for avian species lacking natural resistance (especially those from artic or sub-artic environments) or those hosted in structures like zoos and wildlife rescue centers. With that premise, this study describes the efficacy and safety of a therapeutic protocol to treat avian malaria in three snowy owls (*Bubo scandiacus*) hosted in a wildlife rescue center in Apulia, south of Italy, and affected by avian malaria by *P. relictum*. The protocol consisted of administering 10/4 mg/kg atovaquone/proguanil per os once a day for three consecutive days, repeating this seven days later. Seven days after the end of the treatment, *P. relictum* was not detected in the birds’ blood and no adverse effects were observed during the 60 days of monitoring after the end of the treatment. Therefore, a therapeutic regimen of 10/4 mg/kg/day may be considered safe and effective in a valuable and endangered species such as *B. scandiacus*.

## 1. Introduction

Avian malaria is a ubiquitous disease that affects avian species worldwide, caused by parasites belonging to the genus *Plasmodium* (Apicomplexa: Haemosporida) and transmitted by Culicidae mosquitoes [1]. Their life cycle includes primary and secondary exoerythrocytic merogony stages, which both produce merozoites able to infect the red blood cells (RBCs). Once in erythrocytes, merozoites can evolve in erythrocytic meronts or gametocytes, which may be ingested by mosquitos. Fertilization occurs within the vector, and an ookinete is produced, which will form oocysts that give rise to sporozoites [2]. The infectious stages of the parasite and its pathogenic effects are strongly dependent on the host species, which may be poorly or well adapted to the infection [3]. However, the most recent studies are evidencing a widening of the host range linked to climate change, which contributes to promoting contacts among vectors, parasites, and hosts, thus overturning the preexistent natural, environmental barriers [4]. Within the *Plasmodium* genus, 55 species are known to infect avian hosts [5]. Among those, *Plasmodium relictum* is one the most widespread species, insomuch that it has been classified among the 100 most invasive species in the world [6]. Based on information recorded in the MalAvi database [7], almost 300 bird species have been reported to be infected by one of the five main lineages of *P. relictum*. Such major lineages, namely SGS1, GRW11, GRW4, LZFUS01, and PHCOL01, have been traced according to the nucleotide sequence of the mitochondrial cytochrome b gene (*cytb*) [6]. Lineages have been hypothesized to be a possible factor in the variability observed in the infected birds, which ranges from no clinical signs to death, and that is probably related to the adaptation of the host species and the lineage, although still unknown factors may contribute to the development of the disease [1,6,8]. The more severe forms of acute avian malaria are characterized by vomiting, anorexia, and consequent reduction in body mass, depression, and regenerative anemia due to hemolytic processes [9]. Organs may be affected, and splenomegaly, hepatomegaly, and pulmonary edema have been reported [9]. Acidosis, higher plasma protein concentrations, and reduced hemoglobin oxygen-binding capacity are reported as well, plus low complete blood counts [10]. Neurological signs, such as motor incoordination, convulsions and paralysis, have been observed in penguins [11]. The parasitemia effects on hematocrit vary, harming some bird species while no relevant changes are observed in others [12]. Sudden death was described in penguins [13].

In chronic infections, repeated cycles of damage and replacement of red blood cells have been described, but there has been no correlation found between parasitemia and other parameters such as the oxidative stress and body condition of infected birds. Nevertheless, lower fitness is often associated with the chronic course of the disease [14].

Despite the increasing detection of *Plasmodium* spp. infection in birds and the potentially deleterious effects it may generate in hosts, little knowledge about treatments is available to date. Some studies on chickens infected by *Plasmodium gallinaceum* have described a transient efficacy of chloroquine and doxycycline on the asexual erythrocyte stage, while artesunate and its combination with primaquine were found to be less effective [15]. The combination of atovaquone (AV) and proguanil hydrochloride (PH), commercially known as Malarone^®^, was administered in experimentally infected chaffinches (*Fringilla coelebs*) and greenfinches (*Carduelis chloris*), effecting recovery from infection but a relapse 43 days after treatment [16]. Atovaquone/proguanil was also used to control *Haemoproteus* sp. infection in a goshawk (*Accipiter gentilis*), but details were scarce and the exact dosage was not reported [17].

In light of those considerations, this study aimed to assess the efficacy of AV/PH administered to three snowy owls (*Bubo scandiacus*) at a dose of 10/4 mg/kg.

## 2. Materials and Methods

### 2.1. Case History

One female (SO1) and two male (SO2 and SO3) captive bred snowy owls, aged between 5 and 10 years and weighing about 1.5 kg each, were hosted at the regional wildlife rescue center of Apulia, Italy, under seal, after having been seized because of illegal detention. Each bird was individually housed within large outdoor wire meshed enclosures (length 4 m, width 7 m, height 3 m), in a quiet area far from traffic and adjacent to other birds’ enclosures. The animals were partly live fed, as their diet mostly consisted of live quails, but chicken necks and defrosted chicks were also used.

Several months after their admission, they exhibited anorexia, weight loss, and prolonged bleeding after even minor injuries. Due to clinical concerns, the veterinary staff performed a targeted diagnostic screening to ascertain the potential pathogenic agent.

Avian malaria caused by *P. relictum* was diagnosed by the molecular protocols described in Section 2.2, and the three snowy owls were treated with the combination AV/PH. Specifically, the pharmacological protocol consisted of AV/PH 10/4 mg/kg per os once a day (*sid*) for three consecutive days. After a break of seven days, the administration cycle was repeated. The complete sequence of operations is listed in Supplementary Table S1. Each dose was prepared by properly parceling a Malarone^®^ pediatric tablet (Glaxo Wellcome SA, Aranda de Duero, Spain). To avoid stressful manipulation of the snowy owls and administer the drugs with a meal as recommended to increase absorption [18], each tablet portion was included in a defrosted chick commonly used for feeding the birds. Soon after the diagnosis, to prevent potential contact with insects, the enclosures were covered with small mesh nets.

To assess the efficacy of the treatment and to monitor the health status of the animals, hematological and molecular tests were carried out, according to the schedule shown in Supplementary Table S1. Specifically, about 1.5 mL of blood was collected by expert veterinarians from the ulnar vein of each animal immediately before the first administration of AV/PH (D1), and 7, 30, and 60 days after the conclusion of the therapy (D20, D43, and D73, respectively). One mL of the blood collected from each animal was transferred into a serum separator gel tube (Greiner Bio-One, Cassina de Pecchi, Italy) and 0.5 mL was immediately transferred into a proper lithium heparin tube (Becton Dickinson, Milan, Italy).

### 2.2. Molecular Investigations

Fifty μL heparinized blood underwent total genomic DNA extraction and purification by means of the ZymoBiomics DNA Miniprep Kit (Zymo Research Corporation, Irvine, CA, USA), according to the manufacturer’s instructions. Four μL was used as a template in the first reaction of the nested PCR (nPCR) strategy previously described [19], carried out with slight modifications. Briefly, Platinum II Hot-Start Green PCR Master Mix (Thermo Fisher Scientific, Milan, Italy) was used for the reactions in a final volume of 25 μL, with the primers HAEMNFI (5′-CATATATTAAGAGAAITATGGAG-3′) and HAEMNR3 (5′-ATAGAAAGRTAARAAATACCATTC-3′), designed to amplify a portion of the mitochondrial *cytb* gene of a wide range of apicomplexan blood parasites, at a final concentration of 0.6 μM each. The thermal cycle was the following: 94 °C for 5 min; 35 cycles of 94 °C for 15 s, 52.5 °C for 15 s, and 72 °C for 10 s; and final elongation at 72 °C for 10 min.

Two μL of the first reaction was used as a template in the second reaction, targeting organisms of the genera *Haemoproteus* and *Plasmodium*. The 20 μL final volume of the mixture included the primers HAEMF (5′-ATGGTGCTTTMGATATATGCATG-3′) and HAEMR2 (5′-GCATTATCTGGATGWGATAATGGT-3′) at a final concentration of 0.5 μM each. The thermal cycle was the following: 94 °C for 5 min; 35 cycles of 94 °C for 15 s, 56 °C for 15 s, and 72 °C for 8 s; and final elongation at 72 °C for 10 min.

The PCR products were separated by electrophoresis on a 1.5% agarose gel and visualized by UV exposure after staining in 0.5 μg/mL ethidium bromide. The amplicons obtained in the first step’s PCRs from all the animals were purified and cloned in pTZ57R/T by using the InsTAclone PCR Cloning Kit (Thermo Fisher Scientific), according to the manufacturer’s instructions. The recombinant plasmid was then transformed into CaCl_2_-competent cells of *Escherichia coli* TOP10 and selected as previously described [20]. Five positive colonies per each PCR product, for a total of 15 clones, were selected and the nucleotide sequence of the insert was determined by the Sanger method through a BigDye Terminator v3.1 (Thermo Fisher Scientific) at the Microsynth Seqlab facilities (Göttingen, Germany). The forward and reverse reads were assembled by using Cap3 [21], and the primer sequences were removed. The contigs were aligned among themselves by the ClustalW method, implemented within the CLC Genomic Workbench v. 22.0.1 (Qiagen Digital Insight, Aarhus, Denmark).

Considering they were identical among themselves, only one was submitted to GenBank with the accession number OQ067982 as a representative. The sequence was finally compared with the other ones present in GenBank by the nucleotide BLAST to confirm the identification [22].

### 2.3. Molecular Quantification of Plasmodium relictum

To implement a qPCR to quantify the *P. relictum* load in the birds’ blood, a set of primers and a probe were designed by means of the OligoArchitect tool (Merck, Darmstadt, Germany) [23], on the basis of the nucleotide sequence of the cloned amplicons.

The designed primers and probe cytbqF (5′-CCTTTAGGGTATGATACAG-3′), cytbqR (5′-TTCTGGAACAATATGTAAAGG-3′), and cytbqP (5′FAM-AAATACCCTTCTATCCAAATCT-3′) were purchased from Bio-Rad Laboratories (Milan, Italy) in a premixed 1:1 solution.

The qPCRs were prepared by using the SsoAdvanced Universal Probes Supermix (Bio-Rad Laboratories) in a final volume of 20 μL, the adding primers and probe at a final concentration of 250 nM and 2 μL of the purified DNA solution as a template. The thermal cycle consisted of the polymerase activation at 95 °C for 3 min, followed by 40 cycles of 95 °C for 10 s, 52.1 °C for 15 s, and 60 °C for 15 s, with plate reading. The reactions were carried out in a CFX Connect Real-Time PCR System (Bio-Rad Laboratories) and analyzed by means of CFX Maestro Software v. 2.2 (Bio-Rad Laboratories) with the automatic baseline setting. A standard quantification curve was prepared by serially diluting the purified recombinant plasmid obtained as above described. The initial concentration of the plasmid solution was determined by UV spectrometry and, taking into account the molecular mass of the plasmid and the volume of solution (2 μL) included in each reaction, the target copy number (TCN) was obtained as previously described [24].

Considering that an average 6-fold overestimation has been reported when supercoiled plasmids were used for quantification [25] and that about 20 copies of the mitochondrial chromosome were reported to be present in *Plasmodium falciparum* and *P. gallinaceum* cells [26], the approximated number of *P. relictum* cells per microliter of blood was calculated. Mean values and 95% confidence intervals were calculated and reported in the text. The normal distribution of data was verified by means of the Shapiro–Wilk test and discarded (*p* = 0.011). Therefore, the Mann–Whitney test was used for pairwise comparisons between groups of data, with a significance threshold of *p* = 0.050. All calculations were performed in R v. 4.3.0 [27].

### 2.4. Hematological Investigations

Heparinized blood and serum were used for hematological analyses. Specifically, blood smears were prepared as previously described [28] and the complete blood count (CBC), saline balance, and main enzymatic profile were assessed to ascertain hepatic, renal, and pancreatic functions (Table 1). Each set of data was analyzed for its normal distribution by the Shapiro–Wilk test and the one-way ANOVA was carried out to verify the potential time–response relation of each parameter. Tukey’s honestly significant difference test was applied for pairwise comparison between time points. All calculations were performed in R v. 4.3.0 [27].

## 3. Results

At the diagnosis (D-7), all animals tested positive for *P. relictum*, which persisted up to D1, prior the treatment. The nPCR returned an amplicon of about 600 bp in the first step and an amplicon of about 550 bp in the second step, as expected for positive samples [19]. When the nucleotide sequences of the first step products were determined, they were found to be 571 bp (primers excluded) and 100% identical among themselves. When compared with those present in GenBank, they resulted as being 99.65% identical to a portion of the *cytb* gene of *P. relictum* identified from *Spheniscus demersus* (KY653774), *Loxia curvirostra* (KY653773), and *Corvus corone* (MF189958) [29,30]. Following the molecular detection of *P. relictum*, blood smears were carried out, but the pathogen was not observed. This is not unusual because the level of parasitemia during the infection cycle is quite low, except during the brief erythrocytic merogony stage [1,3,31]. Therefore, blood smears are considered unreliable for diagnosing avian malaria in several species [11,13,32] while, to our knowledge, no specific data are available for snowy owls.

By combining the quantitative data obtained before treatment (at D-7 and D1), the mean concentration of *P. relictum* in blood was found to be 1876 ± 697 cells/μL, with individual oscillations (Table 1). In particular, the average parasitic load in the female SO1 blood was 555 ± 349 cells/μL, significantly differing from the male SO2 (3226 ± 662 cells/μL; *p* = 0.002), but not from the male SO3 (1846 ± 1458 cells/μL; *p* = 0.180). No significant difference was found between the *P. relictum* load of SO2 and SO3 (*p* = 0.149).

Despite a numerical decrease in SO1 and SO3, differences between the pathogen load in blood at D-7 and D1 were not significant (*p* = 0.185).

On D20, 7 days after the last AV/PH administration, all animals tested negative for *P. relictum* during both nPCR and qPCR. Similarly, no amplification products were retrieved at follow-up, on D43 and D73. A further routine check-up carried out about one year after the treatment confirmed the negativity.

The results of hematological tests are reported and listed in Table 2. After the treatment, hemoglobin concentration (HGB) and hematocrit (HCT) values increased significantly (*p* = 0.024 and *p* = 0.012, respectively). Compared with values at D-7, HGB was significantly higher seven days after the treatment (*p* = 0.023) and at D43 (*p* = 0.018) and D73 (*p* = 0.015). Similarly, HCT was higher 30 and 60 days after the treatment with respect to D-7 (*p* = 0.042 and *p* = 0.020, respectively), despite the increase from D-7 to D20 not being significant (*p* = 0.106). No significant variations were observed in renal and hepatic parameters with the exception of γ-glutamyl transferase (GGT), which decreased significantly at D43 and D73 with respect to D20 (*p* = 0.018 and *p* = 0.035, respectively). Unfortunately, the GGT test was not performed at the first sampling.

## 4. Discussion

Avian malaria is a re-emerging threat to the health and welfare of a number of bird species [6]. A wide range of effects, from mild to severe and up to lethal, has been reported [33], as well as its great diffusion potential [34], especially wherever different bird species live in close contact, such as in zoos [32,35]. Promiscuity may be even greater in wildlife rescue centers, which may host vulnerable or endangered species, too. With that premise, the availability of therapeutic options is crucial. However, very few studies are available, mainly because of the difficulty of experimental challenges with wild or unusual bird species.

Studies have been carried out to ascertain the efficacy of doxycycline but, despite exhibiting some prophylactic effects [36], its efficacy in avian malaria therapy has not been proven to be significant [15,36,37]. Additionally, doxycycline is a broad-spectrum antibiotic, and its use should be limited to specific clinical needs to avoid the selection of resistant organisms [38]. Aminoquinolines such as chloroquine and primaquine have been used, alone or in combination, with good results in gyrfalcons for both the prevention or treatment of avian malaria [39], but their severe side effects, which include neurological disorders, do not make them a primary choice [11]. Similarly, pyrimethamine, an inhibitor of the dihydrofolate reductase administered in combination with sulphonamides such as sulfadiazine and sulfadoxine, is effective against *Plasmodium* spp. especially during the vector season, but it is well known to be a teratogen [11,39] and capable of causing severe depletion of the folate reservoirs in vertebrates [39,40].

On the other hand, based on the available data from humans and animals, the combination of AV/PH may be considered among the most effective and safe pharmacological choices for the treatment of avian malaria, at least on a theoretical basis. Atovaquone is a hydroxynaphthoquinone that specifically acts on mitochondrial membranes by interfering with the electron transport chain [41], while proguanil is a biguanide that is metabolized in the host liver to the active metabolite cycloguanil, which acts by inhibiting the dihydrofolate reductase of *Plasmodium* spp. [42]. They are commonly used in prophylactic or therapeutic treatments against malaria in humans, as described by a wide and updated literature. Atovaquone is also used in veterinary medicine to treat protozoal infections, such as toxoplasmosis or piroplasmosis, in dogs or cats, often in association with azithromycin, and it is considered quite safe for animals [43,44]. Actually, AV and PH are known to have a favorable therapeutic index [45], thus making such an association a safe and effective option to treat avian malaria. However, data are needed to set up the dosage and therapeutic protocol, which should balance the benefits and risks of the pharmacological treatment.

Data about the dosage of AV/PH in animals, especially in birds, are mostly centered on the empirical evaluation of the treatment, but sometimes, clear indications of therapeutic regimens are lacking. A previous study reported that goshawks were treated for *Haemoproteus* sp. by administration of half a tablet of Malarone^®^ *sid* for three days, repeated after a week [17]. Despite no data being provided about the weight and status of the treated birds, such a dose can be assumed as quite high (about 83/33 mg/kg/day AV/PH), considering that the average weight of *A. gentilis* ranges from 1 to 1.5 kg.

The protocol described here was based on data from the use of AV/PH in humans in the literature. It is known that the 50% inhibitory concentration (IC_50_) of AV is quite low for susceptible *P. falciparum*, ranging between 0.19 and 4.9 ng/mL [46,47], but the absorption rate of AV is poor after oral administration, with a reduced bioavailability [48]. On the other hand, the high level of AV and PH protein bound (>99% and 75%, respectively) and the lipophilic features of those compounds contribute to reaching a high enough concentration of drugs in the bloodstream and prolonging the effects of the combined drugs [48]. Such parameters, along with clinical studies, have led to establishing a therapeutic protocol of 1000/400 mg AV/PH for the treatment of malaria in humans, corresponding to an average dosage of 14.3/5.7 mg/kg/day, administered *sid* for three consecutive days [46,48]. A similar dosage (13–24/5–10 mg/kg/day) has been applied to children [49].

Considering the above, the protocol consisted of the administration of a daily dose of 10/4 mg/kg AV/PH, lower than those reported, also considering that the owls took AV/PH during a meal rich in fats, a factor known to increase bioavailability and peak concentration up to fivefold [18]. Taking into account the low daily dose, the three-day administration cycle was repeated after a week, to maintain the blood concentration as more stable in the absence of specific pharmacokinetic data, currently not available and not easily collectible due to the ecological value of *B. scandiacus*.

Such a treatment returned an optimal outcome, as no *P. relictum* cell was detected in the snowy owls’ blood seven days after the end of the treatment, and no relapse was observed, even after a year. Moreover, hemoglobin and hematocrit levels significantly improved, suggesting a recovery from the pathological status. Additionally, no side effect was observed. Except for GGT, hepatic and renal biochemical markers did not vary significantly. Despite remaining in the normality range, the blood concentration of the latter significantly decreased at D43 and D73 with respect to D20. It is possible that such values had a slight, transient increase after the treatment and that they rapidly returned to normal values. Those oscillations could not be considered unusual due to the high sensitivity of GGT as a marker, as widely described in humans [50].

The absence of adverse effects in the birds is consistent with the data from humans, for whom association with AV/PH, at even higher doses, is considered safe in adults, children above 5 kg, and pregnant women [51,52,53], but a temporary increase in the hepatic markers has often been observed, with rare events of severe liver injury [54]. Conversely, data about the safety of AV/PH in other animals, and especially birds, are scarce. A recent study carried out on buzzard nestlings evidenced no relevant side effects at doses up to 15/6 mg/kg AV/PH [55]. A previous study hypothesized that AV/PH treatment might induce behavioral modifications, increasing the risk of nest abandonment, but the same authors were doubtful about the association with the pharmacological therapy [56]. Finally, no adverse effects were reported after the administration of high-dose AV/PH in *A. gentilis* [18], confirming that such a drug combination is well tolerated by different avian species.

## 5. Conclusions

All the above considered, the administration of AV/PH 10/4 mg/kg daily for three days, repeated after a week, appears to be effective and safe for the treatment of severe infection by *P. relictum* in the snowy owl *B. scandiacus*. The main limitation of this study remains the low number of treated subjects, but considering that experimental infections or even case-control studies cannot be implemented in such a bird species, considered as valuable and vulnerable by the International Union for Conservation of Nature, these data may be a useful starting point to setting up a suitable therapeutic protocol for the treatment of avian malaria in unconventional, endangered, or exotic species.

## Figures and Tables

**Table 1:** Detection and quantification of *Plasmodium relictum* from the snowy owls’ blood.

Time	SO1		SO2		SO3	
	Nested PCR	Estimated Parasitic Load (Cells/μL Blood)	Nested PCR	Estimated Parasitic Load (Cells/μL Blood)	Nested PCR	Estimated Parasitic Load (Cells/μL Blood)
D-7	Positive	855 ± 138	Positive	2753 ± 825	Positive	3802 ± 1847
D1 ^1^	Positive	254 ± 77	Positive	3700 ± 774	Positive	633 ± 137
D20	Negative	ND	Negative	ND	Negative	ND
D43	Negative	ND	Negative	ND	Negative	ND
D73	Negative	ND	Negative	ND	Negative	ND

^1^ The blood samples were collected before the administration of the first dose of atovaquone/proguanil. SO: snowy owl; ND: not detected.

**Table 2:** Hematological parameters of the snowy owls before (D-7) and after (D20, D43, and D73) the atovaquone/proguanil treatment.

Parameter	Reference Interval	D-7	D20	D43	D73	D-7	D20	D43	D73	D-7	D20	D43	D73	ANOVA *p*
		SO1	SO1	SO1	SO1	SO2	SO2	SO2	SO2	SO3	SO3	SO3	SO3	
RBC (10^12^/L)	2.4–4.7	2.24 ^a^	2.61	2.33 ^a^	2.40	2.76	2.41	2.92	2.54	2.78	2.26 ^a^	2.78	2.37 ^a^	0.683
HGB (g/dL)	NA	12.6	14.4	14.2	14.8	12.6	15.6	16.4	16.3	14.2	15.5	15.3	15.0	0.024 **
HCT (%)	NA	40	43	44	47	40	48	53	52	45	49	48	50	0.081 *
WBC (10^9^/L)	5.1–26.4	8.2	6.0	9.8	5.6	8.0	6.2	7.0	7.4	8.4	5.0 ^a^	6.2	6.4	0.350
Lymphocytes (10^9^/L)	0.3–10.7	5.4	2.8	2.5	2.5	3.7	3.3	2.1	3.6	4.7	2.3	3.0	4.0	0.022 **
Monocytes (10^9^/L)	0.2–1.8	0.3	0.1 ^a^	1.0	0.0 ^a^	0.3	0.2	0.1 ^a^	0.1 ^a^	0.7	0.3	0.1	0.4	0.421
Eosinophils (10^9^/L)	0.1–4.2	0.2	1.1	1.8	0.9	0.6	0.6	1.3	1.0	0.5	1.0	0.7	0.4	0.276
Basophils (10^9^/L)	0.0–0.3	ND	0.2	0.9 ^b^	0.4	ND	0.2	0.0	0.5 ^b^	ND	0.2	0.1	0.4 ^b^	0.322
CPK (IU/L)	20–3338	632	380	316	377	764	317	244	419	692	857	554	422	0.170
AST (IU/L)	171–301	224	230	175	285	269	230	276	323 ^b^	251	303 ^b^	288	309 ^b^	0.294
ALT(IU/L)	NA	27	22	17	23	39	31	36	47	27	33	36	38	0.781
ALP (IU/L)	NA	30	72	46	60	46	45	50	74	76	60	84	66	0.740
GGT (IU/L)	0–5	ND	2.1	0.1	1.2	ND	2.1	1.3	0.1	ND	2.6	0.1	0.9	0.015 *
Bilirubin (mmol/L)	0–3	1.20	2.57	2.57	3.08 ^b^	2.22	2.57	2.22	3.42 ^b^	2.05	2.39	1.20	2.39	0.099
Total protein (g/L)	26–47	34	38	46	35	37	35	33	34	37	40	36	40	0.879
Albumin (g/L)	8–19	12	14	16	12	14	14	14	14	13	15	13	13	0.281
Globulins (g/L)	13–34	22	24	30	23	23	21	19	20	24	25	23	27	0.987
Cholesterol (mmol/L)	3.94–21.70	5.90	7.22	7.06	6.21	7.19	4.68	5.64	5.20	5.66	6.47	7.76	5.61	0.576
Triglycerides (g/L)	NA	0.81	1.07	0.85	0.91	0.91	2.05	0.97	2.26	0.84	1.14	1.04	0.90	0.422
Amylase (IU/L)	151–285	ND	316	318	282	ND	273	210	313	ND	409	233	302	0.260
Creatinine (mg/L)	NA	3.6	4.0	4.5	3.1	4.9	4.0	4.1	3.0	4.7	5.0	4.1	4.1	0.180
Glucose (mmol/L)	12.1–26.0	17.4	19.4	19.7	20.9	17.4	19.8	21.0	19.9	22.1	20.5	18.2	23.0	0.462
Calcium (mmol/L)	1.83–2.78	2.4	2.5	2.7	2.4	2.5	2.6	2.5	2.4	2.5	2.6	2.5	2.4	0.052
Phosphorus (mmol/L)	0.53–2.51	7.3 ^b^	3.2 ^b^	3.1 ^b^	3.1 ^b^	5.3 ^b^	5.8 ^b^	4 ^b^	5.7 ^b^	4.8 ^b^	4.9 ^b^	7 ^b^	4.8 ^b^	0.723
Sodium (mmol/L)	156–170	149 ^a^	160	161	162	159	165	163	163	156	166	152 ^a^	163	0.105
Potassium (mmol/L)	1.8–5.5	1.9	2.2	3.4	1.9	1.8	1.7 ^a^	2.3	1.7 ^a^	2.1	2.3	2.4	2.0	0.076
Chloride (mmol/L)	109–121	107 ^a^	114	115	114	114	115	110	115	117	109	109	115	0.706
Uric acid (mmol/L)	328–1329	410	275	169	269	668	522	442	1041	318	778	1936 ^b^	561	0.827
LDH (IU/L)	132–861	814	481	223	403	601	190	1128 ^b^	553	789	362	362	376	0.366

^a^ Lower than the reference interval. ^b^ Higher than the reference interval. * Significant at 0.05 level. ** Significant at 0.1 level.

## Data Availability

The nucleotide sequence of the amplified portion of the *P. relictum cytb* gene is publicly available at GenBank with the accession number OQ067982. All qualitative and quantitative data are included in this study.

## Supplementary Materials

The following supporting information can be downloaded at: https://www.mdpi.com/article/10.3390/ani13223457/s1, Table S1: Timesheet of the diagnostic and therapeutic actions described in the study.
